# Visualization of Gene Reciprocity among Lactic Acid Bacteria in Yogurt by RNase H-Assisted Rolling Circle Amplification-Fluorescence *In Situ* Hybridization

**DOI:** 10.3390/microorganisms9061208

**Published:** 2021-06-03

**Authors:** Kyohei Horio, Hirokazu Takahashi, Toshiro Kobori, Kenshi Watanabe, Tsunehiro Aki, Yutaka Nakashimada, Yoshiko Okamura

**Affiliations:** 1 Graduate School of Integrated Sciences for Life, Hiroshima University, Higashihiroshima 739-8530, Japan; d203506@hiroshima-u.ac.jp (K.H.); ziphiro@hiroshima-u.ac.jp (H.T.); kwatanabe@hiroshima-u.ac.jp (K.W.); aki@hiroshima-u.ac.jp (T.A.); nyutaka@hiroshima-u.ac.jp (Y.N.); 2 Division of Food Biotechnology, Food Research Institute, National Agriculture and Food Research Organization, Tsukuba, Ibaraki 305-8642, Japan; tkobo@affrc.go.jp

**Keywords:** mRNA, fluorescence *in situ* hybridization, lactic acid bacteria, single cell, symbioses

## Abstract

Recently, we developed an *in situ* mRNA detection method termed RNase H-assisted rolling circle amplification-fluorescence *in situ* hybridization (RHa-RCA-FISH), which can detect even short mRNA in a bacterial cell. However, because this FISH method is sensitive to the sample condition, it is necessary to find a suitable cell permeabilization and collection protocol. Here, we demonstrate its further applicability for detecting intrinsic mRNA expression using lactic acid bacteria (LAB) as a model consortium. Our results show that this method can visualize functional gene expression in LAB cells and can be used for monitoring the temporal transition of gene expression. In addition, we also confirmed that data obtained from bulk analyses such as RNA-seq or microarray do not always correspond to gene expression in individual cells. RHa-RCA-FISH will be a powerful tool to compensate for insufficient data from metatranscriptome analyses while clarifying the carriers of function in microbial consortia. By extending this technique to capture spatiotemporal microbial gene expression at the single-cell level, it will be able to characterize microbial interactions in phytoplankton–bacteria interactions.

## 1. Introduction

Microbes in the environment constitute unique microecosystems and interact with each other. Depending on the circumstances, they often exhibit different proliferation rates between monocultures and mixed cultures. *Sulfurospirillum* cannot grow fermentatively on lactate alone; however, when co-cultivated with another species that relies on hydrogen, the two species can establish a syntrophic interaction and can grow on lactate as the sole carbon source [1]. Gene expression and syntrophic interactions between two species can be clarified using next-generation sequencing (NGS) and genetic attribution; however, microbes in nature are diverse, and similar genes from different organisms are expressed in parallel. This makes it difficult to specify microorganisms with syntrophic interactions. Moreover, most microbes in the environment are well-known as unculturable [2], so NGS data from metagenomic analyses include new sequences derived from unknown species.

Recent advances in NGS analysis enabled us to perform a metatranscriptome analysis with RNA-seq and monitor gene expression in various samples [3,4]. Because this procedure requires RNA extracted from various microbial cells, RNA-seq data provide fragmented RNA sequences that can suggest the closest homology based on a taxonomic information, but they do not reveal the actual species or its function. Data obtained with bulk analyses using mixed mRNA lacks information regarding “who is doing what”. Recent studies revealed that minorities within a microbial consortium played important roles in the consortium’s function, such as methane oxidation, phosphorus removal, and activated sludge [5,6,7,8]. Unfortunately, sequence data from a rare number of microbes tend to be hidden by the large amount of data from dominant species. For these reasons, RNA-seq data alone is insufficient to elucidate the roles of individual microbes, and it will be required for analysis of microbial interactions to visualize the gene expression within the cell of minor species at the single-cell level.

Fluorescence *in situ* hybridization (FISH) has been utilized to visualize individual microbes involved in a consortium based on 16S rRNA, because ribosomes within a cell are abundant enough for labeling. Recently, intracellular mRNA was detected by FISH with signal amplification. Techniques such as single molecule FISH (smFISH) [9,10,11,12] can identify the owner of the mRNA because they specifically and directly detect mRNA *in situ*. In fact, smFISH was used to visualize the spatiotemporal interactions of pathogens with plants and algae [13]. However, existing smFISH protocols require 48 or more oligonucleotide probes that carry the same fluorophore for every single mRNA to satisfy the detection limit; therefore, setting up multiple detection probes is more expensive than other FISH methods and requires longer mRNA sequences than the short reads of NGS data.

We have also developed a new mRNA detection method named RNase H-assisted rolling circle amplification (RHa-RCA). Based on the RCA technique using padlock probe (PLP) and RNase H, it can specifically detect mRNAs of interest without synthesizing cDNA by reverse transcription and generating non-specific noise originating from the DNA sequence. Because this method only requires any 30 bases of an mRNA sequence for PLP recognition, the short and fragmented mRNA sequence data derived from NGS can be used for detection [14]. Moreover, by combining RHa-RCA and FISH, we also developed an *in situ* bacterial mRNA detection method termed RHa-RCA-FISH. This method requires only a single fluorescently labeled detection probe for an mRNA of interest, so that it is less expensive than smFISH and should be advantageous when detecting multiple mRNAs. This method can simultaneously detect the mRNA of interest in both Gram-positive and Gram-negative bacteria, so it could be utilized to visually monitor gene expression in environmental samples containing various bacterial species [15]. On the other hand, because there are a variety of impurities in environmental samples, the ability to detect intrinsic mRNA expression in environmental bacteria has not been well established. Indeed, appropriate bacterial isolation methods are required for environmental samples that often contain enzyme inhibitors. Therefore, we modified RHa-RCA-FISH to visualize the gene expression of interest in a simple consortium whose transcriptome data were already available, and tested whether this method could be applicable to show the gene expression in individual microbes in the consortium.

Yogurt is a simple microbial consortium comprised of two lactic acid bacteria (LAB). *Streptococcus thermophilus* and *Lactobacillus delbrueckii* subsp. *bulgaricus* (*L. bulgaricus*) form a syntrophic relationship that has been well studied [16,17,18,19], and transcriptome data are available [17,20]. As a model consortium, we performed RHa-RCA-FISH against the mRNA of the pyruvate-formate lyase-activating enzyme gene (*pflA*) of *S. thermophilus* in our previous report [15]. According to Sieuwerts et al. [17], *L. bulgaricus* lacks a pyruvate-formate lyase and the enzyme or formate is provided by *S. thermophilus*. Thus, *pflA* is activated in the early logarithmic phase at 4.06-fold in the expression ratio (mixed culture/monoculture) and suppressed in the middle and late phases at 0.47–0.79-fold. However, LAB grow buried in impurities, including protein (casein) precipitates [21,22]. We could only perform RHa-RCA-FISH in the early phase of cultivation because of the large amount of precipitated casein that prevented LAB cell harvest from yogurt.

In this study, we successfully obtained LAB cells at three different cultivation points using a new cell separation procedure. We then demonstrated that RHa-RCA-FISH can be used to visualize the temporal transition of *pflA* in *S. thermophilus* and monitor its expression. d-lactate dehydrogenase (*ldhD1*), a gene expressed throughout the monoculture period, was employed as a marker for *L. bulgaricus.*

## 2. Materials and Methods

### 2.1. Padlock Probe and Detection Probe

The sequences of PLP-*pflA* and detection probes were the same as those described in our previous report [15]. Each PLP-*ldhD1* region is shown in Figure S1, and sequences of the PLPs and detection probes are shown in Table S1.

### 2.2. Real-Time RHa-RCA

PLPs were checked by real-time RHa-RCA using *in vitro* transcribed (IVT) targeted mRNA according to our previous report [14] (Table S2, Supplemental Materials and Methods). The IVT mRNA was mixed with 250 fmol of a PLP in a buffer containing 20 mM Tris-acetate (pH 7.5), 50 mM potassium glutamate (KGlu), and 0.5 mM ethylenediaminetetraacetic acid (EDTA) to a final volume of 10 μL. PLP hybridization was facilitated by incubation at 95 °C for 1 min followed by immediate cooling to 45 °C and incubation for 3 min at 45 °C and 10 min at 30 °C. After hybridization, 10 µL of the ligation mixture containing 1 μL of SplintR^®^ ligase (25 units), 20 mM Tris-acetate (pH 7.5), 20 mM magnesium acetate (MgAc), and 50 mM KGlu was added to the reaction mixture, which was incubated at 37 °C for 10 min to seal the PLP. Finally, 20 μL of a reaction mixture containing 20 mM Tris-acetate (pH 7.5), 10 mM MgAc, 80 mM ammonium sulfate, 10 mM KGlu, 2.0 mM deoxynucleoside triphosphate, 10 mM dithiothreitol, 0.001 units of pyrophosphatase (New England BioLabs, Ipswich, MA, USA), 0.03 units of RNase H (BioAcademia, Ibaraki, Osaka, Japan), 2× concentration of SYBR Green II (Invitrogen, Thermo Fischer Scientific, Waltham, MA, USA), and 200 ng of DNA-free phi29 DNA polymerase (Kanto Chemical, Tokyo, Japan) were added to the ligated mixture, followed by incubation at 30 °C for 2 h to perform the RHa-RCA reaction. For real-time detection, RHa-RCA reactions were performed in a 96-well polymerase chain reaction (PCR) plate, and fluorescence signals were measured every 10 min for 2 h with the FAM filter (excitation wavelength: 482 nm, fluorescence wavelength: 536 nm) of the Thermal Cycler Dice Real-Time System II (TP900, Takara Bio, Otsu, Shiga, Japan). All real-time RHa-RCA experiments were performed in triplicate to estimate the experimental variance. All solutions and mixtures were prepared in a bench-top clean room (KOACH 500F, Koken Ltd., Tokyo, Japan) [23] to prevent contamination that could cause non-specific signals.

### 2.3. Yogurt Fermentation and LAB Collection

Three time points (3.5, 5.5, and 8.0 h) were chosen according to Sieuwerts et al. [17] to compare our results with their microarray data. Figure S2a shows the schematic representation of the LAB fermentation and collection. A mixed culture of *L. bulgaricus* 2038 and *S. thermophilus* 1131 was prepared by fermentation of sterilized non-fat milk (Takanashi Milk Products Co., Ltd., Yokohama, Kanagawa, Japan) using commercial drinkable yogurt (Meiji Bulgaria yogurt, Meiji Holdings Co., Ltd., Tokyo, Japan) as a starter. First, 100 µL of commercial yogurt was inoculated into 10 mL of milk that was prewarmed at 42 °C in a 50-mL conical tube, followed by fermentation for 3.5 h, 5.5 h, and 8.0 h at 42 °C, without shaking. After the addition of 20 mL of RNAprotect Bacteria Reagent (RBR) (Qiagen, Hilden, Germany), cultures were mixed by vortex to remove the proteins in the yogurt and incubated for 5 min at room temperature to stabilize the RNA. The LAB cells were subsequently collected by centrifugation at 10,000× *g* for 10 min at 25 °C, and the cell pellets were then resuspended in a saturated ammonium sulfate (SAS) solution and stored at 4 °C before use. We previously reported that SAS treatment prevents mRNA from degradation and stabilizes it in bacterial cells [15].

### 2.4. Cell Fixation and Permeabilization

LAB cells were fixed and dehydrated using the cell fixation method described previously [15]. After EtOH treatment, the cell pellets were resuspended in 1× TE buffer. Then, a lysozyme solution was added to the cell suspension solution and incubated at room temperature for 10 min to digest the LAB cell walls. Combinations of the amounts of 1× TE buffer and lysozyme solution were as follows: 262.5 µL and 37.5 µL (200 µg/mL) for 7.5 µg-lysozyme, 295 µL and 5 µL (20 mg/mL) for 100 µg-lysozyme, and 250 µL and 50 µL (20 mg/mL) for 1000 µg-lysozyme. After centrifugation at 10,000× *g* to remove the lysozyme solution, the cells were washed twice with 300 µL of 1× phosphate-buffered saline (PBS; 137 mM NaCl, 8.1 mM Na_2_HPO_4_, 2.68 mM KCl, 1.47 mM KH_2_PO_4_, pH 7.4). After centrifugation, the cells were resuspended with 14 µL of UltraPure™ DNase/RNase-Free Distilled Water (Invitrogen) and transferred to a PCR tube to prepare the RCA reaction.

### 2.5. In Situ mRNA Detection Using RHa-RCA-FISH

Figure S2b shows the schematic representation of the RHa-RCA-FISH procedure. *In situ* mRNA detection was performed on permeabilized LAB cells according to the RHa-RCA-FISH method described previously [15]. All solutions and mixtures were prepared in a bench-top clean room [23].

### 2.6. Imaging and Analysis

Images of 4’,6-diamidino-2-phenylindole (DAPI)-stained LAB cells and FISH-labeled LAB cells were taken by a fluorescence microscope (Nikon ECLIPSE Ti2-E, Tokyo, Japan) equipped with a phase-contrast objective CFI PlanApo DM 100 × (Nikon) and an ORCA-Flash4.0 V3 camera (Hamamatsu Co., Shizuoka, Japan). The fluorescence images were taken using an exposure time of 100 msec, and the images were analyzed using ImageJ software (v.1.52a, National Institutes of Health, Bethesda, MD, USA).

## 3. Results

### 3.1. Collection of LAB Cells from Fermented Yogurt

**Figure 1:**
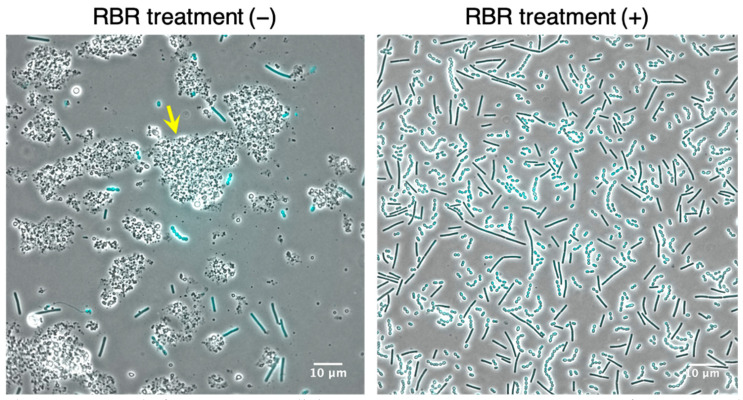
Removal of impurities in milk by RBR treatment. Microscopic images of DAPI-stained LAB cells collected from 8.0 h-fermented yogurt with and without the RBR treatment. Arrow indicates aggregated impurities.

The state of the milk after the addition of yogurt was almost liquid at 3.5 h, semi-solid at 5.5 h, and solid at 8.0 h. Because it was difficult to collect only the LAB cells from solid and semi-solid yogurt, the FISH procedure could only be performed on 3.5-h fermentations, as in our previous report [15]. We therefore developed a method to collect the cells from solid and semi-solid yogurt without impurities. We treated the fermented milk with surfactant-containing RBR before collecting the LAB cells because it helped protect the RNA in cells from degradation during cell collection and concurrently removed the aggregated proteins derived from the casein in milk. Yogurt fermented for 8.0 h was used for this treatment. As a result, many aggregated proteins were found in the collected LAB cells prepared without RBR treatment (Figure 1, left image). In contrast, RBR dispersed the aggregated proteins in the solid state, and the LAB cells were easily collected from the culture solution by centrifugation (Figure 1, right image). This process was also effective for the liquid-state yogurt. Moreover, the RNA in the LAB cells was not degraded with this treatment as confirmed by electrophoresis of the total RNA extracted from cells (data not shown). These results indicate that LAB cells can be easily obtained from yogurt without RNA degradation by RBR treatment.

### 3.2. Establishment of the Amount of Lysozyme Sufficient for LAB Cells Permeabilization

**Figure 2:**
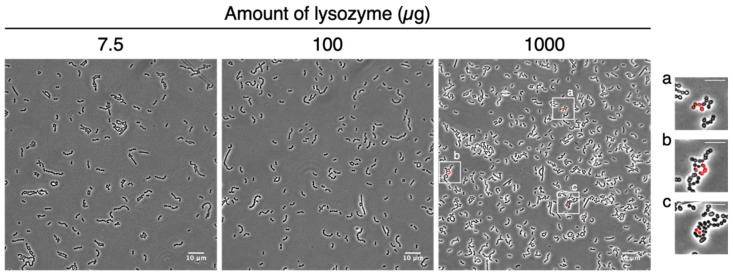
Determination of the lysozyme amount for *S. thermophilus* cell permeabilization. Large images show the detection of *pflA* mRNA using 3.5-h cultivated LAB cells permeabilized with 7.5, 100, or 1000 µg of lysozyme. Images “a” to “c” are magnified images of *S. thermophilus* cells in which fluorescent signals were detected within LAB treated with 1000 µg of lysozyme. Overlays of the phase contrast (grayscale) and Alexa568-labeled probes (red) targeting the RCA products from *pflA* mRNA are shown. Scale bars are 10 µm in the large images and 5 µm in the magnified images.

In our previous report, we successfully extracted total RNA from *Brevibacillus* cells, which were used as a model of the Gram-positive bacteria, using the same procedure at 7.5 µg of lysozyme for *Escherichia coli* as the Gram-negative model bacteria [15]. In contrast, the homogenization of LAB cells (also Gram-positive) was performed by either adding glass beads [17,24] or hard vortex [17,20,24] before total RNA isolation. These reports suggest that the cell walls of LAB cells are stronger than those of the *Brevibacillus* cells. Therefore, we confirmed the optimal amount of lysozyme for permeabilization required for *in situ* detection of LAB mRNA. LAB cells fermented for 3.5 h were treated with different amounts of lysozyme (7.5, 100, and 1000 µg) before performing RHa-RCA-FISH using PLP for *pflA* mRNA (Figure 2). As a result, the bright fluorescent signals that originated from the Alexa568-labeled detection probes were specifically observed from a subset of *S. thermophilus* cells following treatment with 1000 µg of lysozyme (Figure 2, right image), whereas no signal was observed in cells treated with 7.5 and 100 µg of lysozyme (Figure 2, left and middle images).

This result indicates that 1000 µg of lysozyme was suitable for *in situ* mRNA detection in *S. thermophilus* cells from this fermented yogurt. The optimal amount of lysozyme for *L. bulgaricus* was determined to be 100 µg by performing the same experiment targeting *ldhD1* mRNA on 5.5-h fermented LAB cells (Figure S3). We performed subsequent experiments with these conditions using 1000 µg lysozyme for *S. thermophilus* or 100 µg lysozyme for *L. bulgaricus*.

### 3.3. Monitoring of the Temporal Transition of pflA Expression in S. thermophilus Cells by RHa-RCA-FISH

To test whether RHa-RCA-FISH can be used to monitor the intrinsic gene expression of *S. thermophilus* cells collected with the new method, we performed FISH using the collected LAB cells at different fermentation times (3.5, 5.5, and 8.0 h) with selected genes with expression patterns that were confirmed by other methods. We selected *pflA* with an expression pattern that was previously confirmed by microarray [17].

**Figure 3:**
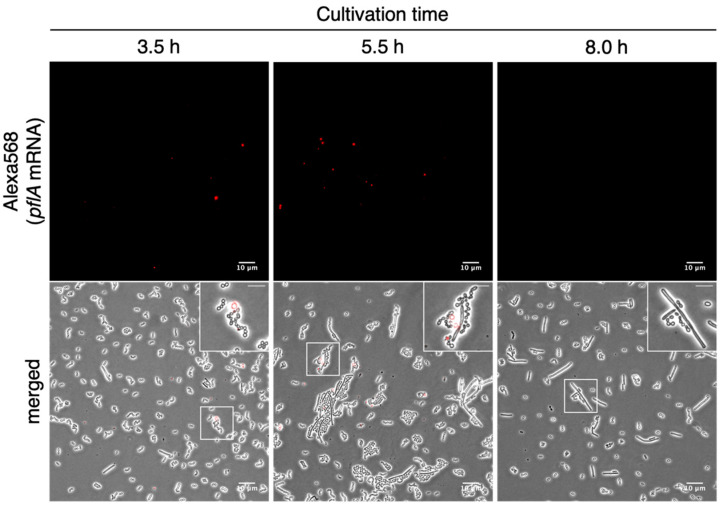
Monitoring of the temporal transition of *pflA* mRNA expression in *S. thermophilus*. Lower images show overlays of the phase contrast (grayscale) and Alexa568-labeled probes (red). Fluorescent signals were difficult to recognize in the merged images because intense light passed through the cells. Therefore, pre-merged images showing fluorescent signals (upper images) are also shown. Insets in the upper right of each merged image are magnified images of the boxes; LAB cells in which fluorescent signals were detected (3.5 h and 5.5 h) and not detected (8.0 h). Scale bars, 10 µm in large images and 5 µm in magnified images.

As a result, the number of *S. thermophilus* cells with fluorescent signals changed over the time course of fermentation; red fluorescent signals were detected in a subset of *S. thermophilus* cells in 3.5- and 5.5-h fermented cells (Figure 3, right and middle images) but not in 8.0-h fermented cells (Figure 3, left images). This result shows that RHa-RCA-FISH could be used to monitor the temporal transition of expression of genes of interest in *S. thermophilus*.

### 3.4. Selection of PLP for Detecting ldhD1 mRNA of L. bulgaricus

**Figure 4:**
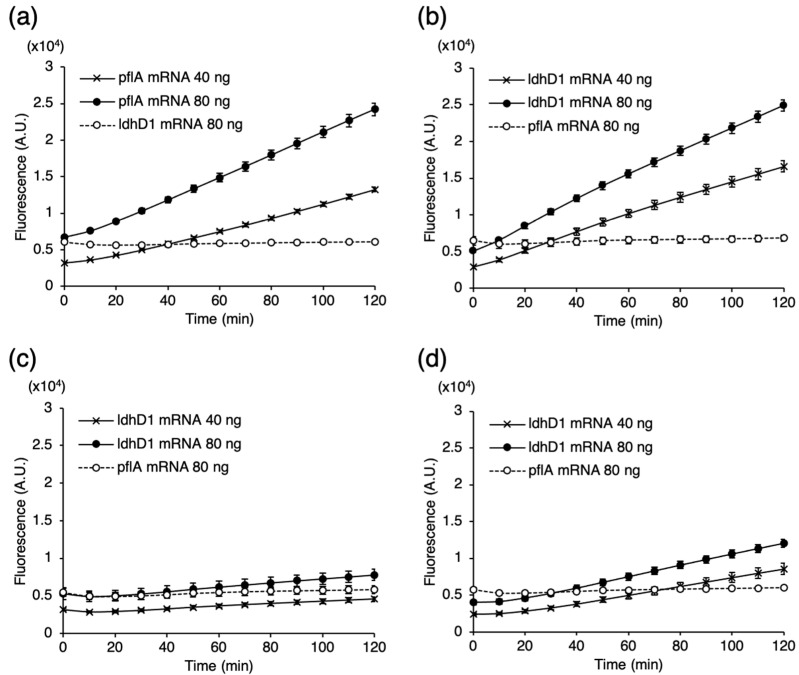
Comparison of the detection sensitivity of PLP-*ldhD1* by real-time RHa-RCA. (**a**) Amplification plots of *pflA* mRNA detection using PLP-*pflA*. (**b**–**d**) Amplification plots of *ldhD1* mRNA detection using PLP-*ldhD1*s; the figures represent Probes #1, #2, and #3, respectively. For each probe, 80 ng of non-target IVT mRNA (*ldhD1* mRNA for PLP-*pflA* and *pflA* mRNA for PLP-*ldhD1*) was used as a negative control.

Expression of *ldhD1* was found in all growth phases [20]. Our preliminary tests using the previous cell preparation procedure failed to detect *ldhD1* mRNA. This could be due to either a low cell collection efficiency or low detection sensitivity of PLP. In any case, we thought that a high-sensitivity PLP was needed to detect *ldhD1* mRNA of *L. bulgaricus in situ*. In our previous report, targeted sequence of PLP set at the middle of the mRNA sequence showed higher sensitivity than that set at the 5′- or 3′-regions [14]. Therefore, we designed three PLPs (#1 to #3) with different targeted sequences in the middle of the *ldhD1* mRNA sequence and compared the amplification plots of the real-time RHa-RCA performed with 40 or 80 ng of IVT *ldhD1* mRNA. Figure 4 shows the amplification plots of the real-time RHa-RCA performed for the target mRNA (solid lines) and non-target mRNA (dashed lines). Probe #1 had the highest signal, which was equivalent to that of the PLP-*pflA* used as the control (Figure 4a,b). The signals observed in Probes #2 and #3 were much lower than that of Probe #1 (Figure 4c,d). This result suggests that Probe #1 has a high sensitivity in the RHa-RCA reaction and is suitable for *in situ* detection of *ldhD1* mRNA.

### 3.5. Monitoring of the Temporal Transition of ldhD1 Expression in L. bulgaricus Cells by RHa-RCA-FISH

To test whether RHa-RCA-FISH can also be used to monitor the intrinsic gene expression of *L. bulgaricus*, we performed FISH in LAB cells by targeting *ldhD1* mRNA with expression patterns previously confirmed by RNA-seq [20]. In our preliminary test, green autofluorescence was observed from residual impurities in the LAB samples, so we employed a new Alexa568-labeled probe (Detection probe-*ldhD1*, Table S1) for *ldhD1* mRNA detection.

**Figure 5:**
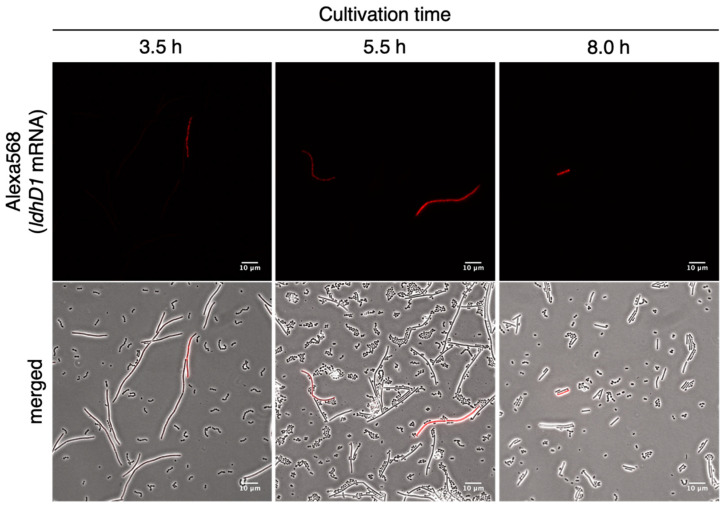
Monitoring of the temporal transition of *ldhD1* mRNA expression in *L. bulgaricus*. The upper images show the fluorescent signals originated from the Alexa568-labeled detection probes; the lower images show overlays of the phase contrast (grayscale) and Alexa568-labeled probes (red). Insets in the upper right of the merged images are magnified images of the boxes. Scale bar, 10 µm.

Fluorescent signals from the Alexa568-labeled probes were specifically detected in *L. bulgaricus* cells in every sample (Figure 5). This result clearly indicates that this method could be used for *in situ* mRNA detection in *L. bulgaricus*. Relatively weak signals were detected in almost all *L. bulgaricus* cells, and multiple strong signals were detected in a subset of whole cells. The number of cells with strong signals did not change significantly throughout the fermentation. This indicates that RHa-RCA-FISH could be used to monitor the temporal transition of the expression of genes of interest in *L. bulgaricus* cells.

## 4. Discussion

We previously reported that RHa-RCA-FISH depends on the cell preparation method [15]. In fact, we had extreme difficulty collecting LAB cells, and only succeeded with mRNA detection from *S. thermophilus* cells collected in the early logarithmic stage of fermentation. We therefore tried to improve mRNA detection from LAB cells with the following approaches: first, seeking a simpler method to remove impurities to increase the amount of collected cells; second, evaluating the optimal amount of lysozyme used for permeabilization; and finally, the examination of the detection sensitivity, especially for *ldhD1* mRNA.

Many methods have been developed to collect LAB cells from yogurt. However, they involve several labor and time-consuming procedures, such as enzymatic treatment [25,26], detergent treatment [25,26], and pH adjustment [17], to remove the proteins in the yogurt. We adopted a simple procedure with RBR to isolate cells from fermented yogurt. This greatly improved the amount of collected cells, and fluorescent signals from the targeted mRNAs were specifically detected in each LAB cell that expressed the mRNAs. Although RBR was used to prevent RNA degradation in several studies that analyzed the transcriptome of LAB [27,28,29], we found that RBR also dispersed the aggregated caseins and facilitated cell collection, even in well-fermented yogurt. This simple and quick method can save time by eliminating complicated procedures for LAB cell collection and will facilitate further progress in LAB morphological studies and transcriptome analyses.

The optimal amount of lysozyme for cell permeabilization was 7.5 µg per reaction in Gram-positive *Brevibacillus* cells in our previous study [15]. However, this study revealed that the optimal amount of lysozyme for LAB cell permeabilization—which are also Gram-positive—was much higher. The optimal amount also differed between the two LAB species. This prevented us from performing simultaneous mRNA detection in the two LAB species, even though we previously reported that different mRNAs could be detected within one RHa-RCA-FISH reaction. This finding indicated that the optimal amount of lysozyme is greatly influenced by the toughness of the cell wall or sensitivity to lysozyme. Environmental samples are composed of a variety of microorganisms, and the toughness of their cell walls vary. Therefore, the present results suggest that the permeabilization method should be optimized for each bacterial sample.

We examined which of the three PLPs showed the best efficiency for *ldhD1* mRNA detection in *L. bulgaricus*, which was not detected in our previous study. Although all PLPs were in the middle region of the *ldhD1* mRNA, there were large differences in their detection sensitivities. This result is likely due to the hybridization efficiency of PLP or the ligation efficiency of the SplintR^®^ ligase, which were affected by the secondary mRNA structure. According to Schneider et al., ligation efficiency was affected by nucleotide pairing of the 5′- and 3′- ends of PLP, the secondary structure of mRNA, and mRNA binding proteins [30]. The secondary structure of mRNA can be predicted *in silico* [31]; however, it requires full-length mRNA sequence data. Because RNA-seq data are fragmented, they are not used for the prediction. Even if genomic data are available, it is not possible to predict the exact secondary structure because information of the 5′- and 3′-untranslated regions of the mRNAs of interest is not yet available. It is therefore necessary to examine the optimal PLP design for each mRNA of interest based on the available information.

This study demonstrated that RHa-RCA-FISH can specifically detect *S. thermophilus* cells expressing *pflA* and *L. bulgaricus* cells expressing *ldhD1* in mixed LAB cells and visualize the temporal transitions in gene expression. Previous transcriptome data showed that *pflA* mRNA was highly expressed in the early phase but decreased toward the late stage of culturing, while *ldhD1* mRNA was constitutively expressed [17,20]. On the other hand, our FISH data show that cells expressing *pflA* mRNA were found in the early phase but not the late phase. Although our FISH data also show that the cells expressing *ldhD1* mRNA were found in all phases, the fluorescence intensity in *L. bulgaricus* cells varied among individual cells. We do not think that there is a major discrepancy between the current FISH data and the previous transcriptome data; rather, it this result strongly suggests individual differences and heterogeneous mRNA expression within a culture. When gene expression was synchronized (e.g., *gfp* transformed to *E. coli* by adding isopropyl β-d-1-thiogalactopyranoside), FISH signals were detected in almost all cells [15]. However, the mRNA expression levels varied among individual cells in samples whose gene expression cannot be controlled (e.g., *Brevibacillus* transformed with *dsred* driven by its native promoter [15] and LAB fermented in milk). In fact, even though gene expression varies in individual cells, transcriptome analysis measures mRNA extracted from bulk cells as the average of the transcription events and cannot clarify such differences [32]. Therefore, it is extremely important to visualize mRNA with FISH to understand gene expression in individual cells. Indeed, in our preliminary experiments performing RT-PCR and real-time RHa-RCA using total RNA extracted from the fermented yogurt, the target mRNAs were below the detection limit (data not shown); however, a few LAB cells showing fluorescence were detected by RHa-RCA-FISH. This suggests that RHa-RCA-FISH can detect small amounts of mRNA in the transcriptome. Moreover, another cell fixation and permeabilization method may improve the signal intensity [33]. Using this FISH approach or an advanced method to visualize the mRNA of minorities *in situ* can reveal their important roles in the microbial consortium. Bulk transcriptome analysis is suitable for observing the activity of the whole microbial consortia but is not suitable for individual cells. *In situ* mRNA detection complements the deficiencies of transcriptome analysis and clarifies the roles of individual cells in microbial consortia.

In conclusion, RHa-RCA-FISH can visualize intrinsic mRNA expression and monitor temporal transitions in gene expression. Its applicability for LAB will help advance research in LAB gene expression during yogurt fermentation. More detailed analyses of the interactions at the single-cell level will contribute to the biochemical characterization of LAB, as well as their breeding and efficient yogurt production. Although we visualized the time course of gene expression involved in the symbiotic relationship of LAB, we could not visualize their interactions. This is because the spatial information of LAB in yogurt was lost with the cell preparation procedure performed to remove impurities. In samples of mammalian tissue [34,35], activated sludge [36], sludge granule [37], biofilm [38], plant [13,39], algae [13], and coral [40], pretreatment procedures that maintain the original location of the sample are used prior to FISH to obtain the spatial information between the host and virus or pathogen. In combination with a new pretreatment procedure that keeps the original location of the bacteria, RHa-RCA-FISH can clarify interactions among microbial consortia. For example, the symbionts within the phytoplankton will be collected as a whole consortium when analyzing gene expression. However, the transcripts from the host phytoplankton might be much more abundant than the symbionts’ mRNA and RNA-seq might not show the gene expression from scarce species. On the other hand, RHa-RCA-FISH can detect the mRNA within the cell at the single-cell level; thus, it would be able to count the number of symbionts within a host cell. By adopting a suitable cell permeabilization method, this approach can be applied to a wide variety of bacteria and will compensate for the insufficiency of RNA-seq data to reveal “who is doing what”.

## Supplementary Materials

The following are available online at https://www.mdpi.com/article/10.3390/microorganisms9061208/s1, Figure S1: Target regions of three PLP-ldhD1s; Figure S2: LAB fermentation and collection, RHa-RCA-FISH procedure; Figure S3: Determination of lysozyme amount for L. bulgaricus cell permeabilization; Table S1: Sequences of PLP and detection probes for pflA and ldhD1 mRNA; Table S2: Sequences of PCR primers for ldhD1; and Supplemental Materials and Methods.
