# Supplementary material for: Visualization of Gene Reciprocity among Lactic Acid Bacteria in Yogurt by RNase H-Assisted Rolling Circle Amplification-Fluorescence In Situ Hybridization
Source: Microorganisms. 2021 Jun 3;9(6):1208. doi: 10.3390/microorganisms9061208 (PMC8228470; doi:10.3390/microorganisms9061208)
Supplement: Supplementary file 1 [file microorganisms-09-01208-s001.zip › microorganisms-1224373-supplementary.pdf]

Visualization of Gene Reciprocity among Lactic Acid Bacteria in Yogurt by RNase H-assisted Rolling Circle Amplification-Fluorescence *in situ* Hybridization

Kyohei Horio, Hirokazu Takahashi, Toshiro Kobori, Kenshi Watanabe, Tsunehiro Aki, Yutaka Nakashimada, and Yoshiko Okamura

Supplemental Figures, Tables, and Materials and Methods

Supplemental Figures and Tables.

1 ATGACTAAAATTTTTGCTTACGCAATTCGTGAAGATGAAAAGCCATTCTT

51 GAAGGAATGGGAAGACGCTCACAAGGACGTCGAAGTTGAATACACTGACA

101 AGCTTTTGACACCAGAAACTGCTGCTTTGGCAAAGGGTGCTGACGGTGTT

151 GTTGTTTACCAACAACTTGACTACACCGCTGAAACTCTGCAAGCTTTGGC

201 AGACAACGGCATCACTAAGATGAGCCTGCGTAACGTTGGTGTTGACAACA

251 TCGACATGGCTAAGGCTAAGGAACTTGGCTTCCAAATCACCAACGTTCCA

301 GTTTACTCACCAAACGCCATCGCAGAACACGCTGCTATCCAAGCTGCCCG

351 CATCCTGCGTCAAGACAAGGCTATGGACGAAAAGGTTGCCCGTCACGACT

401 TGCGTTGGGCACCAACTATCGGCCGTGAAG*TTCGCGACCAAGTTG*TTGGT

451 GTTATAGGTA[#3]CTGGCCACATCGGTCAAGTCT*TCATGCAAATCATGG*AAGG

501 CTTCGGCGCTA[#2]AGGTTATCGCTTACGACATCTTCCGCAACCCAGAATTGG

551 AAAAGAAGGGCTACTA*CGTAGACTCACTTGA*CGACCTGTACAAGCA[#1]AGCT

601 GACGTTATTTCCCTGCACGTTCCTGACGTTCCAGCTAACGTTCACATGAT

651 CAACGACGAGTCAATCGCTAAAATGAAGCAAGACGTAGTTATTGTTAACG

701 TATCACGTGGTCCATTGGTTGACACTGACGCGGTTATCCGTGGTTTGGAC

751 TCAGGCAAGATCTTCGGTTACGCAATGGACGTTTACGAAGGTGAAGTTGG

801 CATCTTCAACGAAGACTGGGAAGGCAAGGAATTCCCAGACGCACGTTTAG

851 CTGACTTGATCGCTCGTCCAAACGTTCTGGTAACTCCACACACTGCTTTC

901 TACACTACTCACGCTGTTCGCAACATGGTAGTTAAGGCCTTCGACAACAA

951 CCTTGAATTGGTTGAAGGCAAGGAAGCTGAAACTCCAGTTAAGGTTGGCT

1001 AA

Figure S1. Target regions of three PLP-*ldhD1*s. The D-lactate dehydrogenase gene (*ldhD1*) sequence of *L. bulgaricus* 2038 was obtained from GenBank under Accession

No. CP000156, locus tag “LBU0066.” Targeted sequences of PLP are highlighted in red, and PLP number are shown as superscripts. Italic sequences are 5'-arm regions, and underlined sequences are 3'-arm regions of PLPs.

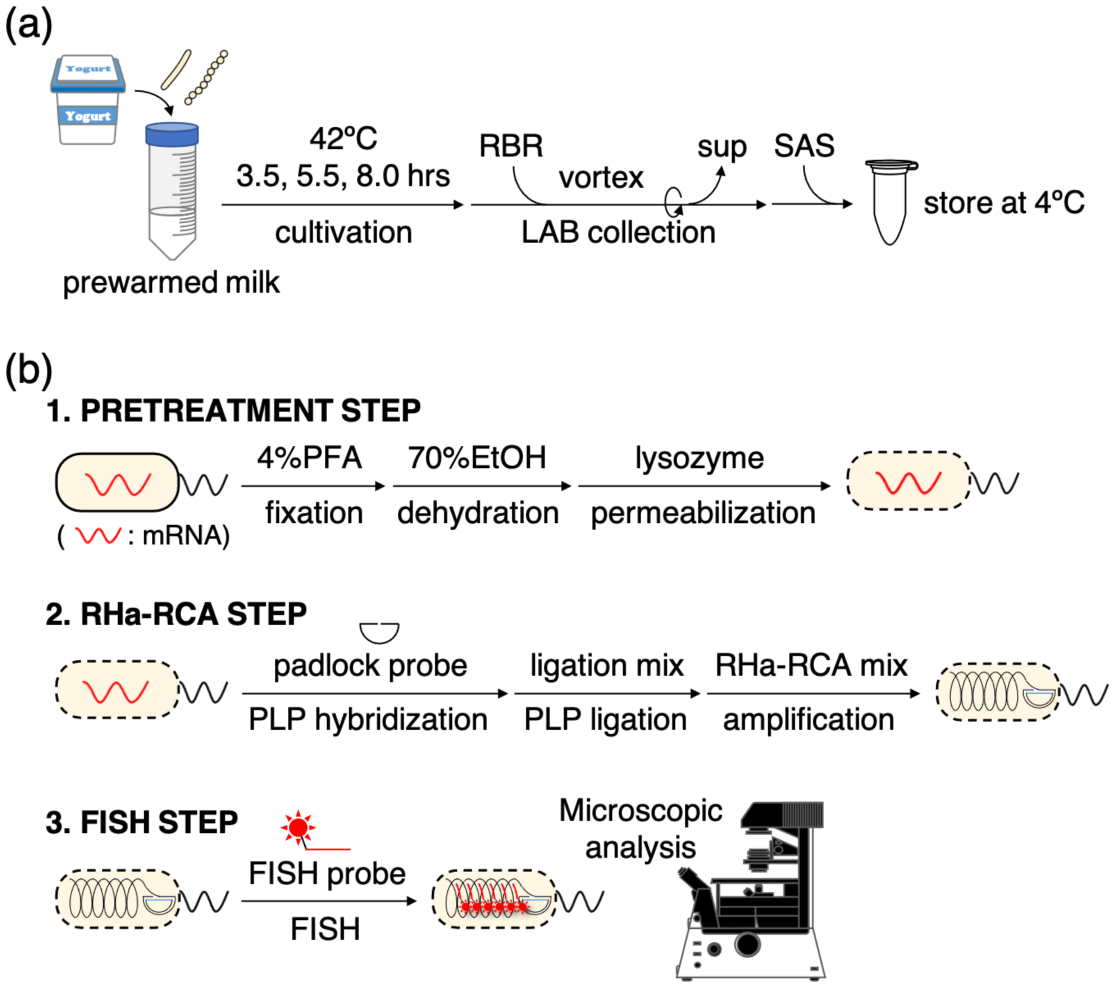

Figure S2. LAB fermentation and collection, RHa-RCA-FISH procedure. (a) Schematic representation of the LAB fermentation and collection procedure. (b) Schematic representation of the RHa-RCA-FISH procedure.

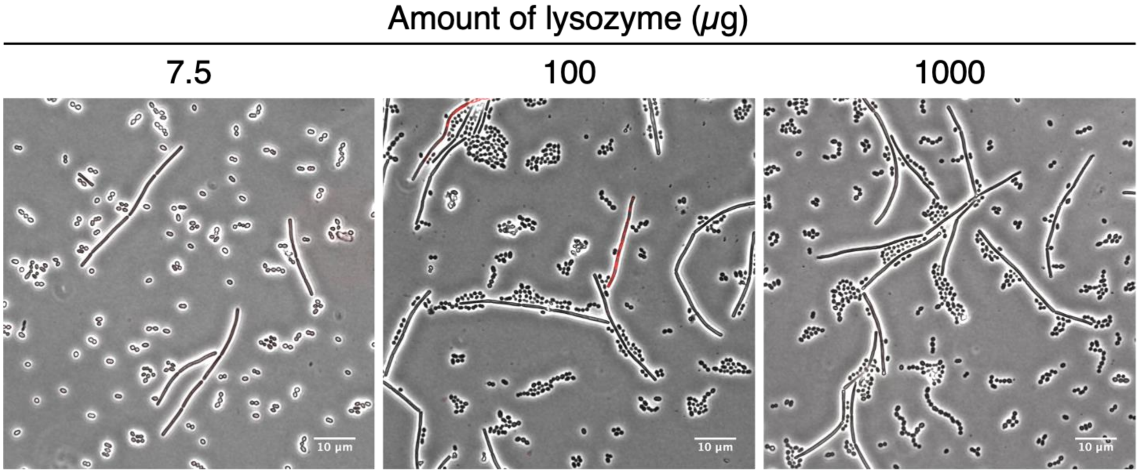

Figure S3. Determination of lysozyme amount for *L. bulgaricus* cell permeabilization. Large images show the detection of *ldhD1* mRNA using 5.5-h cultivated LAB cells permeabilized with 7.5, 100, or 1000 μg of lysozyme. Overlays of the phase contrast (grayscale) and Alexa568-labeled probes (red) targeting the RCA products from *ldhD1* mRNA are shown. Scale bar, 10 μm.

Table S1. Sequences of PLP and detection probes for *pflA* and *ldhD1* mRNA.

Name	Sequence	Length
PLP-*pflA*	5’-*TGTTGTTCGGGGTCA*ttccttttacagCCTCAATGCTGCTGCTGTACTACtcttcCGGGTCACAAACTTG-3’	70 mer
PLP-*ldhD1* #1	5’-*TCAAGTGAGTCTACG*ttcattttacagCCTCAATGCACATGTTTGGCTCCtcttaTGCTTGTACAGGTCG-3’	70 mer
PLP-*ldhD1* #2	5’-*CCATGATTTGCATGA*ttccttttacagCCTCAATGCACATGTTTGGCTCCtcttcTAGCGCCGAAGCCTT-3’	70 mer
PLP-*ldhD1* #3	5’-*CAACTTGGTCGCGAA*ttcattttacagCCTCAATGCACATGTTTGGCTCCtcttcTACCTATAACACCAA-3’	70 mer
Detection probe-*pflA*	5’-Alexa568-CCTCAATGCTGCTGCTGTACTAC-3’	23 mer
Detection probe-*ldhD1*	5’-Alexa568-CCTCAATGCACATGTTTGGCTCC-3’	23 mer

The sequences of PLP-*pflA* and detection probes were the same as in our previous report [15]. The fluorophore of Alexa488-detection probe [15] was changed to Alexa568 in the Detection probe-*ldhD1*. Italic and underlined letters indicate homologous sequences to *pflA* and *ldhD1* mRNA. Uppercase letters indicate detection probe sequence. Sequences of detection probes were from Larsson et al. [41,42].

Table S2. Sequences of PCR primers for *ldhD1*.

Name	Sequence	Length
ldhD1_F_NcoI	GAGAGccatggCTAAAATTTTTGCTTACGCA	31 mer
ldhD1_R_NotI	GAGAgcggccgcTTAGCCAACCTTAACTGGAGTTTCA	37 mer

Lowercase letters indicate recognition sites of restriction enzymes (*Nco*I and *Not*I);

underlined letters indicate homologous sequences to *ldhD1*.

Supplemental Materials and Methods.

Oligonucleotides.

All padlock probes were purchased from Eurofins Genomics (Ebersberg, Germany). Alexa-labelled oligonucleotides were purchased from Japan Bio Services Co., LTD. Each padlock probe region is shown in Figure S1, and the sequences are shown in Table S1. Thiophosphated random RNA hexamer (6R5S, 5'-rN*rN*rN*rN*rN*rN; *, thiophosphate-linkage; rN, random RNA base) [43] was also purchased from Tsukuba oligo service Co., LTD.

All oligomers were dissolved in 0.1× Tris-EDTA (TE) buffer (1 mM Tris-HCl pH 7.5, 0.01 mM EDTA) in a bench-top cleanroom as described above.

Genomic DNA extraction from LAB cells.

First, 8.0-h fermented LAB cells were resuspended in 510 μL of a solution containing 10 mM Tris-Cl (pH 7.5), 0.15 M NaCl, 0.1 M EDTA, and 200 μg lysozyme and incubated at 37 ℃ for 20 min. Next, 67.5 μL of a solution containing 0.5 M Tris-Cl (pH 7.5), 5% sodium dodecyl sulfate, and 100 μg proteinase K was added to the cell suspension. Then,

the genomic DNA was extracted using the phenol-chloroform method, followed by RNase A treatment and purification using the ethanol precipitation method. Genomic DNA concentrations were determined using a Qubit® fluorometer with Qubit® dsDNA BR Assay Kit (Invitrogen/Thermo Fischer Scientific, Waltham, MA), and DNA degradation was confirmed via agarose gel electrophoresis (1%, TAE buffer) and visualized by staining using UltraPower™ DNA/RNA Safe dye and a blue-light transilluminator.

Total RNA extraction from LAB cells.

Total RNA of 3.5-, 5.5-, and 8.0-h fermented LAB cells were extracted using RNAzol® RT (Molecular Research Center, Cincinnati, OH, USA) according to the manufacturer's protocol, followed by DNase treatment. Then, extracted total RNA was purified using a Monarch® RNA Cleanup Kit (New England BioLabs). Total RNA concentrations were determined using a Qubit® fluorometer with Qubit® RNA BR Assay Kits. After denaturation using formamide, a small amount of genomic DNA contamination and degradation of RNA were confirmed in each sample via agarose gel electrophoresis (1.5%,

TAE buffer) and visualized by staining using UltraPower™ DNA/RNA Safe dye and a blue-light transilluminator.

Cell-free cloning of *ldhD1*

The cloning procedure was performed with a cell-free cloning method [43] using phi29 DNA polymerase. At first, full-length *ldhD1* was obtained by performing PCR using a forward primer carrying an 5'-*Nco*I site and reverse primer carrying an 5'-*Not*I site (Table S2). The *ldhD1* fragment was digested by *Nco*I (Takara Bio) with Shrimp Alkaline Phosphatase (SAP, Takara Bio) at 37 ℃ for 1 h following 65 ℃ for 10 min to inactivate enzymes. Then, the *ldhD1* fragment was digested by *Not*I at 37 ℃ for 1 h, followed by purification using a Monarch® PCR DNA Cleanup Kit (New England BioLabs).

The pET-32a (Novagen) was amplified by multiply-primed rolling circle amplification (MPRCA) [44] using an RNA primer (6R5S) [43]. The amplified pET-32a was digested by *Not*I (Takara Bio) with SAP at 37 ℃ for 1 h following 65 ℃ for 10 min to inactivate enzymes. Linearized pET-32a was digested by *Nco*I, then isolated by agarose gel electrophoresis and purified with Wizard® SV Gel and PCR Clean-Up System

(Promega, Madison, WI, USA).

The *ldhD1* fragment and vector were ligated using T4 DNA ligase (Takara Bio) in 1× CutSmart buffer (New England BioLabs) with 1 mM ATP at 16 ℃ for 30 min following 65 ℃ for 10 min to inactivate enzymes. To remove the linear DNA, the ligated product was treated with Plasmid Safe ATP-Dependent DNase (Epicentre, Madison, WI, USA) and Exonuclease I (New England BioLabs) in 1×CutSmart buffer with 1 mM ATP (final conc.) at 37 ℃ for 4 h following 80 ℃ for 20 min to inactivate enzymes. Then the remaining circular ligated products were amplified with 6R5S and DNA-free phi29 DNA polymerase [45] (Kanto Chemical) at 37 ℃ for 16 h following 65 ℃ for 10 min to inactivate enzymes according to our previous report [43]. Cloning success or failure was confirmed by restriction enzyme digestion and agarose gel electrophoresis.

***In vitro* transcription of *ldhD1* mRNA**

In vitro transcription of *ldhD1* mRNA was performed using a T7 RiboMAX™ Express Large Scale RNA Production System (Promega) and linearized pET-*ldhD1* by *Not*I digestion according to the manufacturer's protocol. After RNase-free DNase I (New

England BioLabs) treatment, the transcribed mRNA was purified using a NucleoSpin® RNA Clean-up XS kit (Macherey-Nagel) according to the manufacturer's protocol. RNA concentrations were determined using a Qubit® fluorometer with Qubit® RNA BR Assay Kit. The transcribed mRNA was stored at -80 ℃ until required.

References

41. Larsson, C.; Koch, J.; Nygren, A.; Janssen, G.; Raap, A.K.; Landegren, U.; Nilsson, M. *In situ* genotyping individual DNA molecules by target-primed rolling-circle amplification of padlock probes. *Nat. Methods* **2004**, *1*, 227–232, doi:10.1038/nmeth723.

42. Larsson, C.; Grundberg, I.; Söderberg, O.; Nilsson, M. *In situ* detection and genotyping of individual mRNA molecules. *Nat. Methods* **2010**, *7*, 395–397, doi:10.1038/nmeth.1448.

43. Takahashi, H.; Yamamoto, K.; Ohtani, T.; Sugiyama, S. Cell-free cloning using multiply-primed rolling circle amplification with modified RNA primers. *BioTechniques* **2009**, *47*, 609–615, doi:10.2144/000113155.

44. Dean, F.B.; Nelson, J.R.; Giesler, T.L.; Lasken, R.S. Rapid amplification of plasmid and phage DNA using Phi29 DNA polymerase and multiply-primed rolling circle amplification. *Genome Res.* **2001**, *11*, 1095–1099, doi:10.1101/gr.180501.

45. Takahashi, H.; Yamazaki, H.; Akanuma, S.; Kanahara, H.; Saito, T.; Chimuro, T.; Kobayashi, T.; Ohtani, T.; Yamamoto, K.; Sugiyama, S.; *et al.* Preparation of Phi29

DNA Polymerase Free of Amplifiable DNA Using Ethidium Monoazide, an Ultraviolet-Free Light-Emitting Diode Lamp and Trehalose. *PLoS ONE* **2014**, *9*, e82624, doi:10.1371/journal.pone.0082624.
